# Self-Powered Photodetector Based on FTO/*n*-TiO_2_/*p*-CuMnO_2_ Transparent Thin Films

**DOI:** 10.3390/ma15155229

**Published:** 2022-07-28

**Authors:** Carmen Lazau, Mircea Nicolaescu, Corina Orha, Viorel Şerban, Cornelia Bandas

**Affiliations:** 1 National Institute for Research and Development in Electrochemistry and Condensed Matter Timisoara, 300569 Timisoara, Romania; carmen.lazau@gmail.com (C.L.); nicolaescu.mircea13@yahoo.com (M.N.); orha.corina@gmail.com (C.O.); 2 Department of Materials and Manufacturing Engineering, Faculty of Mechanical Engineering, University Politehnica of Timisoara, 300222 Timisoara, Romania; viorel.serban@upt.ro; 3 Romanian Academy of Technical Sciences, 300223 Timisoara, Romania

**Keywords:** self-powered photodetector, *n*-TiO_2_/*p*-CuMnO_2_ heterojunction, thin film

## Abstract

A self-powered photodetector with the FTO/*n*-TiO_2_/*p*-CuMnO_2_ configuration, representing the novelty of the work, was successfully achieved for the first time and presumes two steps: deposition of the *n*-type semiconductor (TiO_2_) by the doctor blade method and of the *p*-type semiconductor (CuMnO_2_) by the spin coating technique, respectively. Investigation techniques of the structural and morphological characteristics of the as-synthesized heterostructures, such as XRD, UV-VIS analysis, and SEM/EDX and AFM morphologies, were used. The *I-t* measurements of the photodetector showed that the responsivity in the self-powered mode was 2.84 × 10^7^ A W^−1^ cm^2^ and in the 1 V bias mode it was 1.82 × 10^6^ A W^−^^1^ cm^2^. Additionally, a self-powered current of 14.2 nA was generated under UV illumination with an intensity of 0.1 mW/cm^2^. Furthermore, under illumination conditions, the response time (t_res_) and the recovery time (t_rec_) of the sensor exhibited a good response; thus, t_res_ = 7.30 s and t_rec_ = 0.4 s for the self-powered mode, and in the 1 V bias mode, these were t_res_ = 15.16 s and t_rec_ = 2.18 s. The above results show that the transparent heterojunction device of *n*-TiO_2_/*p*-CuMnO_2_ exhibited a self-powered ultraviolet photodetector with high sensitivity.

## 1. Introduction

Self-powered ultraviolet photodetectors (SPVs) have been widely studied lately because they have a great advantage; namely, they do not need any external power sources, can operate continuously and independently and do not require high costs to produce them [1]. The operating principle of photodetectors (PD) is very well known, being based on the conversion of photon energy into an electrical signal by photogenerated carriers [2,3,4]. The PD performance is mainly dependent on the properties of the semiconductor materials used, regarding their abilities to transform optical signals into electrical signals that are determined by a process of electron-hole pair generation and recombination within semiconductor materials [5]. Self-powered operation happens because of the photovoltaic effect, i.e., the photocurrent generated without applied bias, and requires a heterojunction to be formed in the device. In most cases, the dark current has high values, and thus power losses appear, this being a major disadvantage, leading to unwanted power losses [6]. In this way, to remove the mentioned inconveniences, UV photodetectors have been studied and developed that have the ability to detect UV radiation and produce an electric field, so they can be self-powered. 

Over time, it was shown that metal oxide heterojunctions can facilitate photovoltaics for self-powered operation due to their built-in potential, low cost, non-toxicity and easy synthesis methods [6]. Thus, a self-powered UV photodetector based on a *p*-*n* heterojunction has a main advantage due to its built-in potential that automatically separates generated electron-hole pairs [7]. Therefore, various metal oxide semiconductor materials (i.e., TiO_2_, ZnO, CuO and NiO) have been applied for different types of photovoltaic cells [8,9], but from them, TiO_2_ was selected as the most desirable material for photovoltaic devices [10]. B. Yin and coworkers developed an ultraviolet detector based on the FTO/TiO_2_/MoO_3_ heterojunction with potential well-trapping electrons under dark and demonstrated the excellent responsivity under UV illumination [11]. Furthermore, transparent photovoltaic cells and self-powered photodetectors were fabricated by a TiO_2_/NiO heterojunction, with an excellent UV response in the photovoltaic mode and high transparency in the visible light region (about 57%) [12]. Z.M. Bai et al. reported a UV self-powered photodetector based on a ZnO nanowire array in which a high sensitivity of 475 without external bias was found [13]. Y. Xie and coworkers fabricated a self-powered UV detector based on single-crystalline rutile TiO_2_ nanorod arrays grown directly on FTO and demonstrated a fast photoresponse speed, high photosensitivity, excellent spectral selectivity, low-cost fabrication and environmentally friendly characteristic [14]. Over time, copper-based delafossite-type metal oxides CuMO_2_ (M = Mn, Fe, Cr, Al, Ga, In, B) have offered excellent properties that have attracted a great deal of attention, especially CuMnO_2_, due to their excellent optical and electrical characteristics [15,16,17]. C.-L. Hsu et al. successfully obtained the configuration *p*-CuMnO_2_/*n*-ZnO NW with excellent organic gas sensing and UV and visible light detection [18]. A study by Y. Zhang et al. demonstrated a self-powered UV photodetector based on heterojunction-type CuSCN/ZnO nanorods, with excellent stability and reproducibility characteristics [19]. R.R. Prabhu et al. reported the obtaining of a highly transparent and high conducting *p*-CuO/*n*-ZnO heterojunction, with applicability in the development of efficient and low-cost optoelectronic devices, particularly photodetectors, solar cells and gas sensors [20]. 

Based on our previous research in the field of the development of TiO_2_-CuMnO_2_ thin films obtained in different configurations and demonstrating the functionality of heterojunctions as well as the ability to detect UV radiation [21,22], the progress and novelty of this work is related to the design and fabrication of a self-powered and transparent UV photodetector with the FTO/*n*-TiO_2_/*p*-CuMnO_2_ heterojunction. A completely solution-processed transparent (>60%) *p*-*n* heterojunction has been fabricated in the structure FTO/*n*-TiO_2_/*p*-CuMnO_2_ and the performance of the photodetector under dark and UV illumination conditions is investigated. Finally, an increased performance during the self-powered mode (zero bias) as well as in the normal bias mode in terms of high responsivity, good recovery time and high sensitivity in 0 V bias was exhibited by the UV photodetector device.

## 2. Materials and Methods

### 2.1. Chemicals

All reagents were of analytical purity grade and used without further purification, as follows: titanium isopropoxide (TTIP, 98%), α-terpinol, ethyl cellulose, acetone, ethyl alcohol, fluorine-doped tin oxide (FTO, 99%), Cu(NO_3_)_2_·3H_2_O, Mn(NO_3_)_2_·4H_2_O, sodium hydroxide were purchased by Sigma-Aldrich Company.

### 2.2. Fabrication of FTO/n-TiO_2_/p-CuMnO_2_ Heterojunction Photodetector

**Figure 1:**
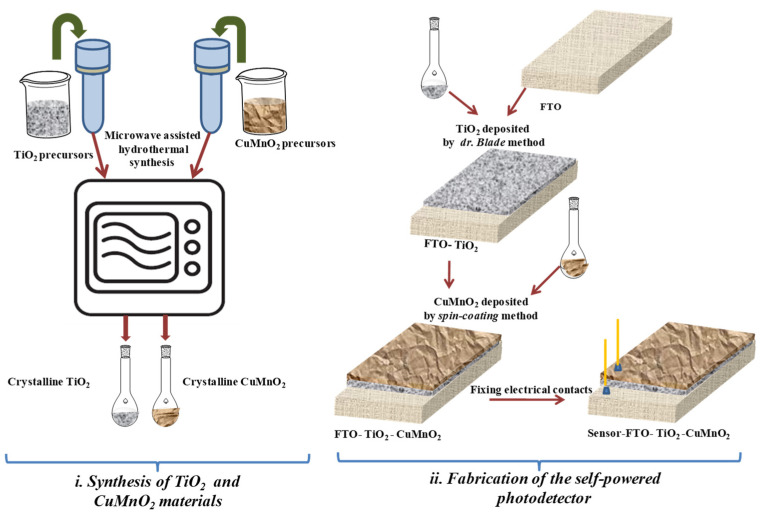
Schematic illustration of self-powered photodetector fabrication.

The self-powered photodetector was achieved in several steps, as presented in the schematic diagram in Figure 1.

(i) Initially, TiO_2_ and CuMnO_2_ powders were synthesized by the microwave-assisted hydrothermal method. Therefore, for TiO_2_ synthesis, the solution was obtained by mixing 40 mL of distilled water (DI) with 6 mL of TTIP under continuous stirring for 2 h. The solution was then introduced into a quartz autoclave with 50% degree of fullness and treated in a Multiwave 300 (*Anton Paar, 2.45 GHz*) microwave reaction system for 30 min at a temperature of 200 °C. On the other hand, the CuMnO_2_ nanocrystalline compound was obtained as previously reported [23]. The CuMnO_2_ powder used in this research was treated for 5 min at 180 °C in a microwave reaction system. After autoclaving, the synthesized samples (TiO_2_ and CuMnO_2_) were filtered, washed with distilled water and dried at 80 °C for 24 h. For thin film deposition, the TiO_2_ and CuMnO_2_ solutions were prepared, according to the following protocol: 0.2 g of TiO_2_ powder and 0.1 g of CuMnO_2_, respectively, were mixed with a matrix solution consisting of solution of ethylcellulose and α-terpinol. For a good homogenization, both solutions were placed in the ball mill (*Lab Mills lx QM vertical planetary ball mill*) at a frequency of 40 kHz for 14 h.
(ii) The next step was to deposit the TiO_2_ and CuMnO_2_ films on the FTO support. Therefore, the FTO was cleaned with acetone, ethanol and DI water in an ultrasonic bath, followed by treatment for 20 min in UV ozone cleaner (*Ossila Producer*). The deposition of the thin and transparent TiO_2_ film on the FTO support was achieved with the conventional doctor blade method, using the previously obtained TiO_2_ solution. The as-obtained structure, FTO-TiO_2_, was dried for 30 min to 60 °C, and calcinated at 450 °C for 1 h. The last step, the deposition of the thin and transparent CuMnO_2_ film on the FTO-TiO_2_ structure, was achieved, to fabricate the UV photodetector. Deposition of CuMnO_2_ thin films using the spin coating method (*WS-400-6NPPB Spin Coater, Laurell Technology Corporation*) presumes the mixing of a homogenized CuMnO_2_ solution with ethyl alcohol and deposition twice for 30 s with a speed rotation of 4000 rpm. For the removal of the organic compounds used in the homogenization solution matrix, and to facilitate the adhesion of the CuMnO_2_ film to the TiO_2_ film, a thermal treatment of 250 °C was applied for 1 h. The collection of electrical data was performed by means of metal wires affixed with silver paste. One wire was placed on the FTO and the other on the CuMnO_2_ film.

### 2.3. Materials and Electrical Characterization

The crystalline structure of the films was characterized by X-ray diffraction (*XRD, PANalytical X’Pert PRO MPD Diffractometer, Almelo, The Netherlands*) with Cu-Kα radiation in the range *2theta* = 20–80°. The morphology of the layers and films was examined using scanning electron microscopy (*SEM, FEI Inspect S model, Eindhoven, The Netherlands*), coupled with the energy dispersive X-ray analysis detector (EDX) for elemental analysis of the as-synthesized samples. Atomic force microscopy (*AFM, Model Nanosurf^®^ EasyScan 2 Advanced Research, Liestal, Switzerland*), with non-contact mode (scan size of 1 µm × 1 µm,) was used to determine particle sizes on the surface, surface roughness and topographical parameters *Sp* and *Sv*, necessary for the calculation of thickness films. The optical properties of the as-synthesized samples were recorded using UV-VIS analysis (*PerkinElmer Lambda 950 UV/Vis spectrophotometer, Shelton, CT, USA*) with an integrating sphere, in the range of 300–600 nm. The band gap *Eg* of the semiconductor materials was determined by plotting the Kubelka–Munk function against energy (eV).

Electrical measurements of the obtained photodetectors were performed using the Keithley 2450 SourceMeter SMU Instruments (*Keithley Company, Cleveland, OH, USA*). I-V measurements were performed to test the functionality in dark and UV illumination of the “*n*-*p*” heterojunction between *n*-TiO_2_ and *p*-CuMnO_2_. The measurements were made in direct polarization at a voltage between −1 V and 1 V with a step rate of 10 mV/s. Ultraviolet detection characteristics were evaluated by recording the current as a function of time, both in 0 V self-power mode and in 1 V power mode in the dark and under lamp illumination at 365 nm with a light power of 0.1 mW/cm^2^. The calibration of the UV lamp power was performed using a Solar 4000 sensor device (AMPROBE) (*Octopart Inc., New York, NY, USA*).

## 3. Results and Discussion 

### 3.1. Structural and Morphological Properties

**Figure 2:**
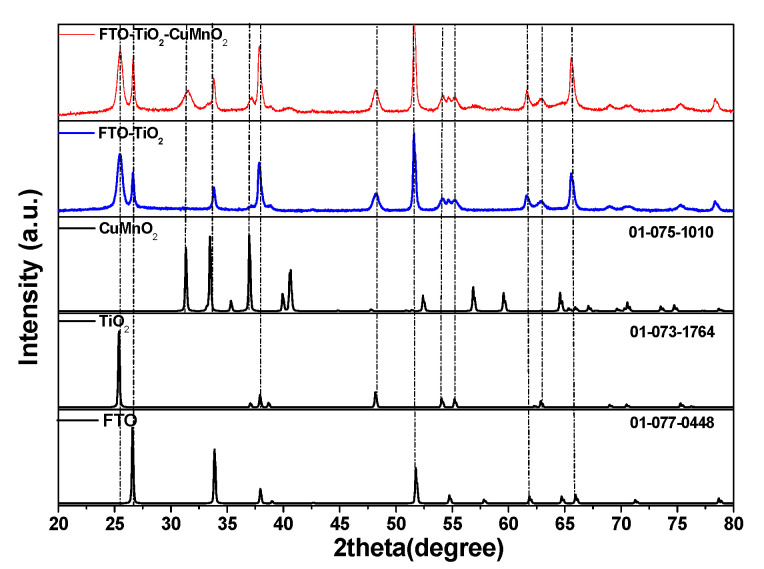
X-ray patterns for the FTO-TiO_2_-CuMnO_2_ heterostructures.

Figure 2 shows the XRD spectra for the FTO-TiO_2_ film and the as-synthesized FTO-TiO_2_-CuMnO_2_ films. The results presented show that microwave-assisted hydrothermal treatment for TiO_2_ synthesis was determined to obtain a rutile crystalline form, confirmed by the main peaks at *2theta*: 27.65°, 32.47°, 54.64° (JCPDS 01-073-1764). For the CuMnO_2_ powder, the XRD pattern confirms the crednerite phase identified by the main peaks at *2theta*: 31.32°, 33.45°, 37.0°, 52.42°, 56.85° (JCPDS 01-075-1010) [23]. Additionally, the main peaks for FTO were identified at *2theta*: 26.67°, 33.90°, 37.96°, 51.84°, 61.87°,65.85° (JCPDS 01-077-0448). Based on X-ray analysis, the average crystallite sizes were calculated by the Debye–Scherrer formula and were estimated to be about 20.80 nm for TiO_2_ and 11.10 nm for CuMnO_2_, respectively. In the XRD pattern of the FTO-TiO_2_-CuMnO_2_ heterostructure, the peaks referring to the phases of CuMnO_2_ and TiO_2_ are preserved, and the successful synthesis of the crystalline *n*-TiO_2_ / *p*-CuMnO_2_ heterojunction is confirmed.

**Figure 3:**
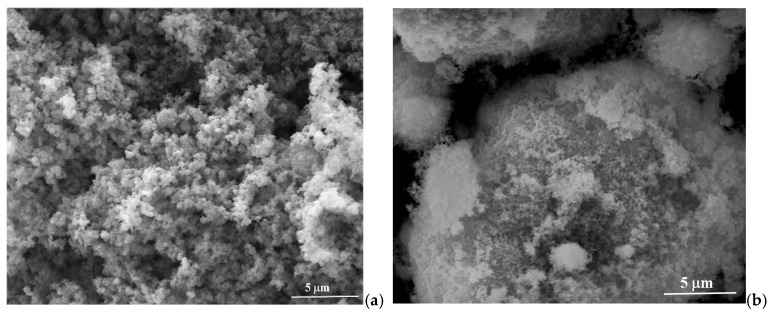

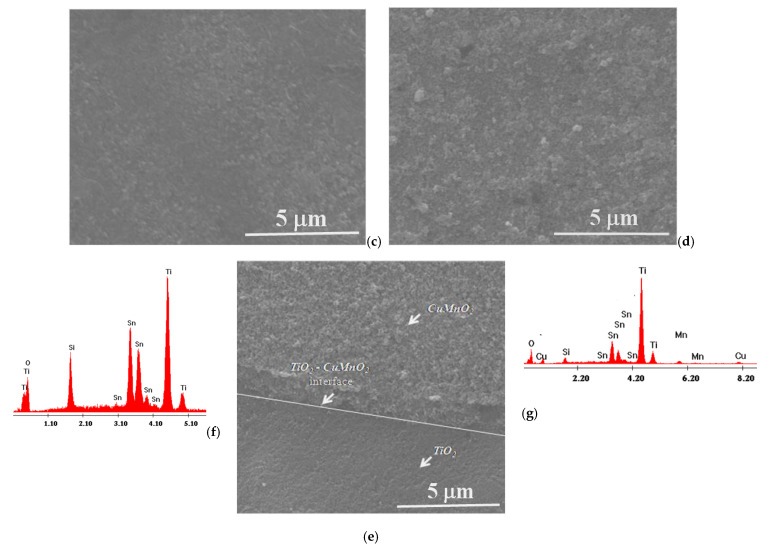
SEM images of the TiO_2_ (**a**) and CuMnO_2_ powders (**b**); TiO_2_ (**c**) and CuMnO_2_ films (**d**); interface TiO_2_-CuMnO_2_ films (**e**); EDX spectra of the FTO-TiO_2_ (**f**) and FTO-TiO_2_-CuMnO_2_ (**g**) structures.

The SEM images of the TiO_2_ (Figure 3a) and CuMnO_2_ (Figure 3b) powders exhibited a spherical morphology for TiO_2_ nanoparticles, and homogenous and uniformly crystals for CuMnO_2_ pristine, respectively. Figure 3c–e presents the surface morphologies for TiO_2_ film (Figure 3c), CuMnO_2_ film (Figure 3d) and the interface between the TiO_2_ and CuMnO_2_ films (Figure 3e). From the analysis of the SEM images, it can be evidenced that the TiO_2_ film is deposited uniformly on FTO, without agglomeration and cracks (Figure 3c). Moreover, the CuMnO_2_ film is deposited uniformly on FTO-TiO_2_ and presents the same morphological characteristics as the TiO_2_ film (Figure 3d). EDX analysis (Figure 3f,g) confirms the presence of chemical elements: Si, Sn, Ti and O from the FTO-TiO_2_ structure (Figure 3f), and Si, Sn, Ti, O, Cu and Mn from the FTO-TiO_2_-CuMnO_2_ structure, respectively (Figure 3g).

**Figure 4:**
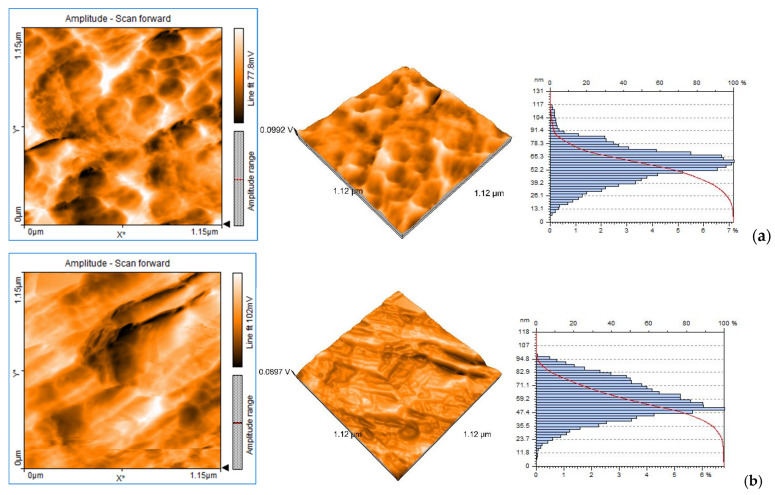
Two-dimensional and 3D AFM surface images and particle size distribution for FTO-TiO_2_ film (**a**) and FTO-TiO_2_-CuMnO_2_ film (**b**).

The surface morphologies by AFM analysis for the structures FTO-TiO_2_ and FTO-TiO_2_-CuMnO_2_ are shown in Figure 4a,b. AFM surface morphologies confirm the results obtained by the related SEM images, for both structures. For the determination of the particle sizes on the surface films, Nanosurf EasyScan2 software was used. The particle sizes determined by AFM and XRD techniques are in accordance with each other, and the size ratio is preserved, but with some differences due to the different calculation algorithms used for each technique. The surface roughness was measured on a surface area of approximately 1.326 pm^2^ and was determined from the values of *Sa* (average roughness) and *Sq* (the mean square root roughness). Topographical parameters *Sp* (the maximum peak height deviation of the roughness) and *Sv* (the maximum valley depth deviation of the roughness) were used for the calculation of the layers’ thicknesses [22,24,25]. All the obtained data are presented in Table 1.

### 3.2. Optical and Electrical Characteristics

**Figure 5:**
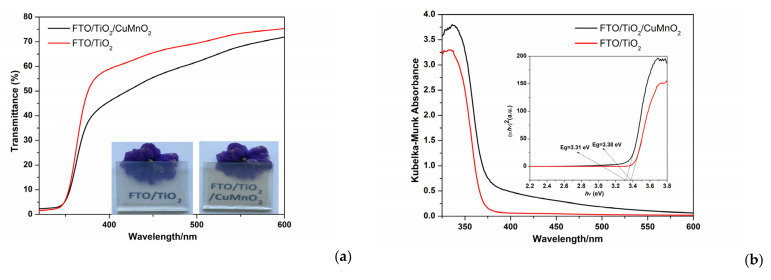
Transmittance spectra and pictures of FTO-TiO_2_ and FTO-TiO_2_-CuMnO_2_ transparent films (**a**); *Kubelka–Munk* absorption and Tauc’s plot for *Eg* calculation (**b**).

To confirm the transparency characteristic of the heterostructure devices, UV-VIS spectroscopy analysis was used for FTO-TiO_2_ and FTO-TiO_2_-CuMnO_2_, as shown in Figure 5a,b respectively. The UV-VIS spectra indicate the transparency of the devices in the visible light region, which is attributed to the wide band gap of TiO_2_ (3.38 eV) and CuMnO_2_ (3.31 eV). The light transmittance pattern decreases when a thin film of CuMnO_2_ is deposited on top, with an average visible light transparency of 60%, 10% lower than for the FTO-TiO_2_, this being generated by the increase in the total thickness of the layers (Figure 5a). According to Nguyen et al., a 40% transparency requirement for applications in window-type sensors must be accomplished by films [12]. In this way, both as-synthesized films demonstrate an average transmittance higher than the 40% transparency requirement.

In Figure 5b, a maximum absorption region at near 330 nm can be seen and presents a decrease at around 385 nm wavelengths for both samples. This is due to the strong absorption spectra of TiO_2_ film and *n*-TiO_2_/*p*-CuMnO_2_ heterojunction attributed to the electron transition from the valence band to the conduction band. This points to the high absorption selectivity of UV radiation for sensors and power generation.

**Figure 6:**
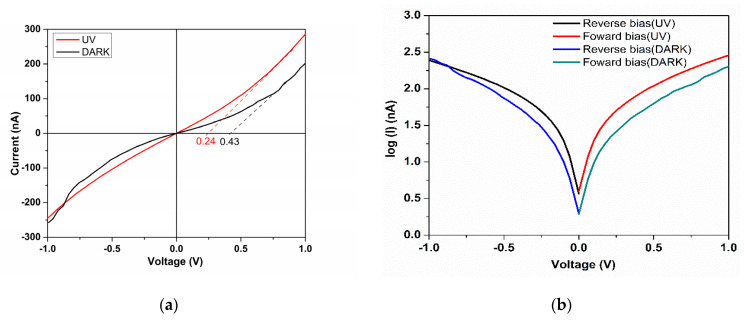
Current–voltage characteristics of the transparent heterojunction of *n*-TiO_2_/*p*-CuMnO_2_ under dark and UV illumination (**a**); *Log* of forward and reverse bias under dark and UV-illuminated conditions. (**b**).

The current–voltage measurement in the dark and under UV illumination is presented in Figure 6a, when an increase in current in the forward bias by the UV illumination from 196 nA (DARK state) to 283 nA (UV state) was generated. Moreover, under UV illumination, a reduction in the turn-on voltage is shown, almost half from the dark state of 0.43 V to 0.24 V. This aspect confirms that the heterojunction under UV illumination decreases the power consumption of the junction.

The *I-V* characteristics of TiO_2_ and CuMnO_2_ thin films deposited on the FTO substrate with silver contact confirm the rectifying nature of the *n-p* junction, and an increase in the asymmetry between the forward and reverse biases in photo illumination suggests a higher rectification behavior under UV exposure.

To evaluate the *I-V* characteristics of the *n-p* junction, the thermionic emission current–voltage relation was used, expressed by Equation (1).

*I* = *I*_0_ [exp (*V*/*n k T*) − 1] (1)

where *T* is the temperature in Kelvin (298 K), *k* is the Boltzmann constant (1.6 × 10^−19^ C), *n* is the ideality factor, *V* is the applied voltage, and *I*_0_ is the reverse saturation current. The reverse saturation current (*I*_0_) and the ideality factor (*n*) can be calculated from the intercept and slope of the straight-line region of the forward bias in the *Log (I)–V* plot, presented in Figure 6b. Based on the literature data, a previous report on the TiO_2_/CuMnO_2_ heterojunction has shown that the ideality factor is between 3.95 and 6.48 depending on the process parameters of the experimental program [21,22]. The heterojunction parameters of FTO-TiO_2_-CuMnO_2_ under both dark and UV illumination conditions are presented in Table 2, where *I_F_* and *I_R_* are the current under forward and reverse bias, respectively, and *V_T_* is the turn-on voltage. The ideality factor *n* = 5.58 is greater than its normal range (1 < *n* < 2) [26]. Considering that thermionic emission is responsible for carrier recombination at the junction but does not drive the transport mechanism, it can be argued that this is determined by defects of the quasi-neutral zone and the junction [27].

**Figure 7:**
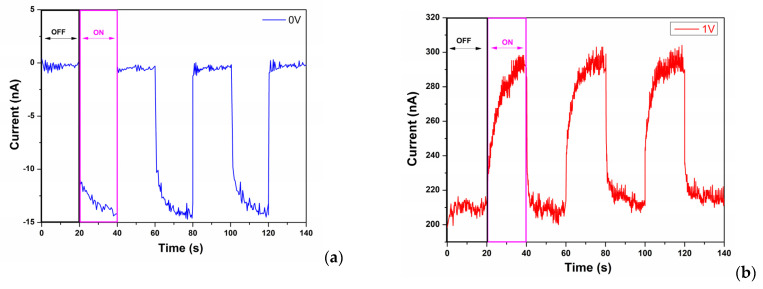
UV time-dependent photoresponse properties of the sensors. (**a**) Response of the sensor in self-powered mode; (**b**) UV response of the sensor at 1 V bias voltage.

The photocurrent measurement in the dark for the self-powered mode was approximately −0.3 nA, and when the photodetector was illuminated for 20 s with a UV light intensity of 0.1 mW/cm^2^, a self-powered current of 14.2 nA was generated; the results are presented in Figure 7.

The sensitivity of the photodetector at the 0 bias was calculated from the ON/OFF ratio (*I_UV_/I_DARK_*) of approximately 48.3, much higher than for the 1 V bias when it was detected as approximately 1.4 [28]. Responsivity values were calculated using Equation (2) [29]

*R = (I_UV_−I_DARK_)*/*P_opt_ S* (2)

where *I_UV_* is the photocurrent under UV lighting, *I_DARK_* is the current in the dark, *S* is the effective area (2 cm^2^) and *P_opt_* is the optical power of the UV source (0.1 mW/cm^2^). From Equation (2), the responsivity value of the thin film TiO_2_ / CuMnO_2_ heterojunction photodetector in self-powered mode is 2.84 × 10^7^ A W^−1^ cm^2^, and in 1 V bias mode it is 1.82 × 10^6^ A W^−1^ cm^2^.

The response time was calculated from the time taken to increase from 10% to 90% of the photocurrent, and the recovery time from the time taken to decrease from 90% to 10% of its maximum value [4]. For the self-powered mode, the response time is 7.3 s and the recovery time is 0.4 s, and in 1 V bias mode the response time is 15.16 s and the recovery time is 2.18 s.

**Figure 8:**
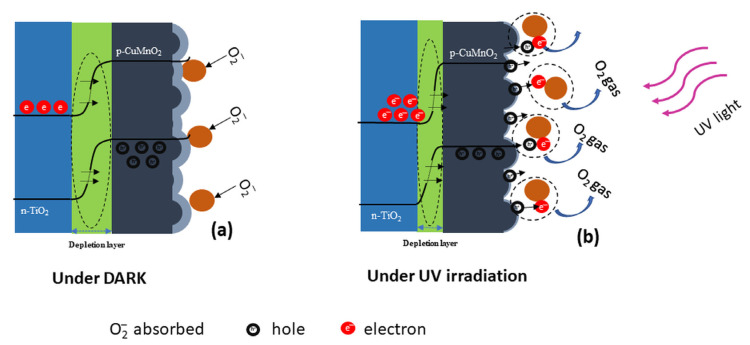
FTO-TiO_2_-CuMnO_2_ in darkness (**a**); FTO-TiO_2_-CuMnO_2_ in UV irradiation (**b**); band diagram of the FTO-TiO_2_-CuMnO_2_ device at 0 V (**a**) in darkness and (**b**) under UV irradiation.

Although junctions built in two-dimensional layers have a theoretical surface area smaller than junctions built in one-dimensional or mixed layers, because the size of both the TiO_2_ and CuMnO_2_ crystallites is very small, both of the contact surfaces at the interfaces between oxides and with O_2_ (gas) and O_2_^-^ (ads) increase the effective active area at a level almost similar to other techniques. This higher surface area increases electron hole pairs generation without applying an external current (0 V bias) to the FTO-TiO_2_-CuMnO_2_ photodetector [1]. The higher oxygen absorption–desorption process is facilitated by the crednerite layer due to the small nanosize of the crystals and the relatively rough surface obtained after deposition [23]. Figure 8 presents a schematic representation of the detection process under dark (Figure 8a) and ultraviolet (UV) (Figure 8b) irradiation conditions for a bilayer heterostructure device. Furthermore, in Figure 8a,b, the band diagram of the current conduction mechanism at the surface interface of the CuMnO_2_ and TiO_2_-CuMnO_2_ junction interface under dark and illumination conditions at 0 V bias is presented.

In the dark state, oxygen atoms from the atmosphere interact with the free holes presented in *p*-type CuMnO_2_ and the adsorbed oxygen ions, and due to the high surface area, oxygen molecules are adsorbed on the surface of the crednerite layer, which introduces a potential barrier (Figure 8a). Thus, a high-conductivity hole accumulation region is formed on the surface of the CuMnO_2_ layer, according to the following Equation (3) [18]

O_2_ (gas) ↔ O^−^ (ads) + h^+^ (3)

This generates a downward band of the energy band on the surface of *p*-CuMnO_2_, as shown in Figure 8a. The light absorption process occurs under UV illumination (Figure 8b) when the photon energy generated is greater than the band gap value. Some of the photons generated by UV illumination are absorbed by photosensitive semiconductors and the generated energy excites the electron in the valence band and is launched to the conduction band; this generates many electrons hole pairs in the heterojunction interface of the TiO_2_ layer, and the photogenerated holes go to the CuMnO_2_ surface [12]. These holes are trapped by adsorbed oxygen molecules on the surface of CuMnO_2_ and, therefore, the desorption process happens, releasing oxygen and narrowing the depletion zone and lowering the heterojunction interface. The photogenerated recovery of O_2_^−^ to O_2_ (g) is represented by the following Equation (4) [18]

O_2_^−^(abs·) + h^+^ → O_2_ (gas) (4)

Therefore, due to the high specific surface area of CuMnO_2_, an increased number of oxygen molecules are adsorbed and, therefore, a larger number of photogenerated holes are trapped by the adsorbed oxygen on the surface of CuMnO_2_. The electron hole recombination process is narrowed, and a greater separation of the charge carriers is generated, resulting in a good photoconductivity of the photodetector device [12]. The self-powered photovoltaic device based on *n*-TiO_2_/*p*-CuMnO_2_ heterojunction is transparent, also due to the broad energy band gap of the metal oxide semiconductor used as presented in Figure 5.

## 4. Conclusions

A self-powered photodetector with the configuration FTO/*n*-TiO_2_/*p*-CuMnO_2_ was successfully achieved for the first time, to the best of our knowledge, by using a layer-by-layer technique and doctor blade and spin coating methods. The structural and morphological characteristics of the FTO/*n*-TiO_2_/*p*-CuMnO_2_ structures confirmed the phases’ stability and purity as well as being uniformly covered, without cracks of the synthesized thin and transparency films. Due to the alignment of the band and the transparency of the layers, a device based on the *n*-*p* junction was demonstrated to operate without an external voltage bias. The device shows an excellent ON/OFF ratio at 0 bias of about 48.3, much higher than that for the 1 V bias of about 1.4. Moreover, good responsivity values under low ultraviolet illumination in both modes was calculated. The response and recovery times are relatively slower for both modes due to the slow mobility of the photogenerated holes in the *n*-*p* depletion layer; instead, when the 0 V bias is applied, these times improve and are faster. The above results show that the transparent heterojunction device of *n*-TiO_2_/*p*-CuMnO_2_ exhibited a self-powered ultraviolet photodetector with high sensitivity.

## Figures and Tables

**Table 1:** Surface particle size, nano-roughness and layer thickness.

Sample	Particle Size (nm)	*Sa* (nm)	*Sq* (nm)	*Sp* (nm)	*Sv* (nm)	Layer Thickness *Sp-Sv* (nm)
FTO-TiO_2_	65	18.99	23.948	85.265	−77.617	162.882
FTO-TiO_2_-CuMnO_2_	47	24.452	31.068	82.229	−109.28	191.509

**Table 2:** Electrical parameters of the FTO-TiO_2_-CuMnO_2_ photodetector.

Sample	Type	*V_T_* (V)	*I_F_* (A)	*I_R_* (A)	*n*	*I_0_* (A)
1 V bias FTO-TiO_2_-CuMnO_2_	Dark	0.43	196 × 10^−9^	257 × 10^−9^	5.58	1.35 × 10^−9^
	UV	0.24	283 × 10^−9^	245 × 10^−9^	-	1.90 × 10^−9^

## Data Availability

Not applicable.
